# Dreaming is not enough. Audiovisual methodologies, social inclusion, and new forms of youth biopolitical resistance

**DOI:** 10.3389/fsoc.2022.1020711

**Published:** 2022-12-05

**Authors:** Paula Guerra, Sofia Sousa

**Affiliations:** ^1^Faculty of Arts and Humanities, Institute of Sociology of the University of Porto, Iscte, Centre for the Study of Socioeconomic Change and the Territory, Transdisciplinary Research Centre-Culture, Space and Memory, Griffith Center for Social and Cultural Research, University of Porto, Porto, Portugal; ^2^Faculty of Arts and Humanities, Institute of Sociology of the University of Porto, University of Porto, Porto, Portugal

**Keywords:** NEETs, arts-based research, youth-led participatory research, Cerco neighborhood, biopolitics of social inclusion, digital cinema, citizenship, community inclusion

## Abstract

The eleventh Sustainable Development Goal, “make cities and human settlements inclusive, safe, resilient and sustainable,” can only be truly answered when there are no individuals in our societies who feel forgotten by the various social institutions. Not in Education, Employment, or Training [NEET] are among those most affected by this social invisibility. Nevertheless, these young people are not alienated or lost. Far from it. Instead, some of them found in the arts registered in the community—music, dance, photography and graffiti—a possibility to resist the various social stigmas attached on them. This was the view on which we conducted our artistic and social intervention, based on the innovative “arts-based research” methodology and “youth-led participatory research,” called “The Neighborhood is Ours II!,” with young NEETs in the socially underprivileged Cerco neighborhood of Porto in Portugal in 2022. We propose a theoretical-empirical approach around a visual/narrative sociology—namely using digital cinema—which will be based on a short film about the life narrative of a young NEET, who has used artistic practices to establish himself in the city of Porto as a cultural mediator. Thus—through these processes of co-creation of knowledge (cine-making)- we aim to demonstrate how the use of the arts can be a key tool in promoting social inclusion and reducing/minimizing feelings of insecurity, but also act as a means of resistance to the daily adversities experienced by marginalized young people and, of course, demonstrate the ways in which the use of artistic practices plays a pivotal role in the development of sustainable and alternative professional, social futures and citizenship.

## The dialogical complexity between spaces, actors, and resources

Social exclusion is neither in territories nor in youngsters, but rather in societies: in the way they structure themselves and link the several dimensions of social exclusion with others, namely economic, social cultural, political, etc., such as market exchanges, redistribution of wealth and reciprocity (Guerra, 2002; Sousa, 2018). How youngsters relate and interact with these dimensions determines the extent to which the population fringe remains integrated in society or not. This is the premise for this article, in view of which we will have as a key issue the connection between the concept of young NEET (Not in Employment, Education or Training) and the potential of arts-based research and social interventions.

In this article, we intend to analyse—in a critical and reflexive way—a initiative developed in the framework of the Workshops called 'The Neighborhood is Ours! We will focus on the short film that was made during the workshops with (and about) an artistic mentor, but also a partner and young resident of the Cerco do Porto Neighborhood^1^—Ricardo Lopes aka Ricardinho^2^. The initiative “The Neighborhood is Ours!” was based on a set of artistic workshops—music, creative writing, photography and graffiti—in one of the most problematic and stigmatized social neighborhoods of the city of Porto, in Portugal. The Workshops took place between the months of May and June 2021. As far as our role as researchers is concerned, it is important to highlight that during the Workshops, we participated in a formative way, in the sense that we carried out the artistic activities provided together with the young people and the artistic mentors, that is, with *Ricardinho* and other members of the OUPA!^3^ group. Thus, the adoption of this posture by the research team provided the establishment of close relationships, sharing and above all trust with the young people; a relationship that remains active through social networks, by sharing music, projects, or ideas, as well as being directly related to the project's objective: to establish a sociological analysis based on prevention in action. As the workshops unfolded, it became evident the need to leave an audiovisual record of the trajectory of *Ricardinho*—one of the rappers of the OUPA! group, the president of the OUPA! association and our partner—a young NEET who saw in the arts an escape and a weapon. Thus, we considered it of utmost relevance to report, in the first person, the story of a young NEET, hence our focus, in this article, on the short film “Dreaming is not enough” that was made in the scope of this initiative.

To offer the reader some background about *Ricardinho* and our partnership relationship, we felt it was important to briefly recount some turning points. Let's see that the first contact we made with *Ricardinho* was by phone in 2018. We sent him a message, in which we asked if he would be interested in being interviewed for a master's project (Sousa, 2018) to which he replied in the affirmative. The first meeting took place on a Thursday morning, around 10 a.m., in Bairro do Cerco do Porto where he was born and currently lives. Ricardo took us to the community studio of the OUPA! Group and Association, which he was part of as an artist and as president of the Association. The interview lasted 3 h and was followed by a guided tour of the neighborhood. This meeting took place in 2018, 5years ago, when Ricardo was 25 years old. The several meetings since then and the countless conversations allowed the creation of a relationship, as well as fostered a bond with the neighborhood, making us feel an integral part of it. This framework, from our point of view, is extremely relevant. And the same goes for *Ricardinho*'s life story. Today, *Ricardinho* is 30 years old and we know his aspirations, dreams, desires, projects, representations about the neighborhood, OUPA! and society. Therefore, when we started our activities in the neighborhood in June 2021, the first thing that came to our minds when we met *Ricardinho* was to make a documentary about him, OUPA!, the neighborhood, life and dreams. Finally, it remains to make a brief note about the production method of the short film. Keeping in mind Ricardo's passion for the arts and the need to do something different, it is not surprising that the invitation came from him. His main goal was to have a visual and sound record of what he is and his visions for the Cerco do Porto Neighborhood and its young people. Thus, a partnership was created between researchers, audiovisual technicians and Ricardo, and the editing, recording, directing and editing process was carried out in a participative logic, with no hierarchies, but rather partnerships. This horizontality as a working method materialized in specific results, such as the obtaining of artistic techniques by the researchers, for example. The research was made from the action. Theory came later.

In recent decades, there have been several studies that address the temporal extension of youth (Bennett, 2013; Way, 2020). In an era of compulsory schooling, entry into the labor market also happens at a later stage. This creates possibilities for greater experimentation in the trajectories of the youngest, but on the other hand, it extends the time of their dependency. The very entry into the labor market, as a consequence of the advanced marginality, the entry of these young people into the labor market is difficult and fragmented, marked by a series of constraints, the most glaring example being the fact that they have to hide their place of residence so as not to be excluded from the selection and hiring processes (Pais, 2012). Viera et al. (2018) warn that we cannot just look through the prism of unemployment or difficulties in labor access. The young population today is characterized by issues as varied as intermittent trajectories, with short-term jobs followed by periods of training or inactivity, school dropouts or unwanted training, etc. (Brooks, 2009; Ferreira T. et al., 2017; Ferreira V. S. et al., 2017).

To account for this complex reality, Eurostat has introduced the concept of NEET (not in education, employment or training) at the beginning of the 2000s. This concept considers young people who are not in employment, education, or training, and is therefore broader in scope than the concept of youth unemployment (Maguire, 2015). For the Portuguese reality, the NEET youth rate has been falling and is currently at 11%, below the European average, which stands at 13.7%. However, this still implies that 181 thousand Portuguese young people were neither working nor in education or training in 2020. If we draw on the contributions of Holte (2017, 2021), we can find some clues that are crucial for us to understand the NEET phenomenon, going beyond a mere statistical description. In his work, Holte (2017) begins by referring to the difficulties he experienced in finding NEET young people when carrying out his research work, something that is similar to our case and the Portuguese reality, because it is extremely difficult to pinpoint an exact number of NEETs (Guerra et al., 2022), since most of these young people are not registered in databases, platforms or institutions that could provide statistical data of this nature.

For the Portuguese reality, the NEET youth rate has been falling and is currently at 11%, below the European average, which stands at 13.7%. However, this still implies that 181 thousand Portuguese young people were neither working nor in education or training in 2020: 44% between 20 and 24 years old and 45.1% between 25 and 29 years old, which proves what was previously mentioned. If there are no significant differences between boys and girls, there is a difference of almost 4 percentage points between young people born in Portugal (10.8%) and young people born outside Portugal (14.5%) (GEP, 2022).

This approach is not without its critics. For Furlong (2006), the NEET concept only covers up the main problem for young people: unemployment (Istance et al., 1994). For others, it encompasses very different realities, from truly disadvantaged young people to young people who, for example, decide to take a gap year (Avis, 2014). Moreover, it is a top-down concept and there is a big gap between the objective and the subjective dimension, i.e., how young people identify (or not) with this new category. Quite simply, young people may not identify themselves as being NEET, considering that they are carrying out activities, understood by them as labor occupations, but which are not understood as such by Eurostat (Holte, 2017), such as new working arrangements linked to the gig economy (Wall et al., 2015) or DIY trajectories (Guerra, 2017, 2018).

This fits in with the reality of today's neoliberal societies, in which there is a great appreciation of the entrepreneurial skills of younger people, such as resilience or self-employment. For McRobbie (2018), this vocabulary of entrepreneurship is not always accessible to young people from more disadvantaged social classes. On the other hand, Willis (1990) argues that young people are very relevant cultural producers and consumers, who use their cultural resources to create their own cultural production. This author postulated the concept of common culture, which has a double meaning: first, it can be found anywhere and is shared between individuals and groups. Thus, young people would use their symbolic creativity through cultural media and new forms of digital technology to be active cultural producers.

To explain this reality, Howard (2022) resorts to two theoretical concepts. First, subcultural capital (Thornton, 1996), which refers to youth cultures and can be defined as participation in non-traditional cultural practices, such as DIY cultures, digital arts (Guerra, 2022b), among others. Second, entrepreneurial capital (Firkin, 2018), which refers to learning techniques and values that can be converted into jobs, usually as freelancers or linked to cultural industries: self-promotion, branding, networking, etc. (Haenfler, 2018). Thus, dispositions are form when young people invest in social fields and, in this way, promote actions, experiences and meanings.

It is remarkable how little NEETs, in Portugal, are studied academically (Cairns et al., 2014; Rowland et al., 2014; Guerra, 2022a). If on the one hand there is this absence of studies, we have, on the other hand, a huge concern in the application of participatory methodologies, such as art-based research, participatory action research, positive youth development, cine-worlding, among others, to this social group, with the concern of enhancing greater community participation and seeing these disadvantaged young people as resources, for themselves and for their communities.

Speaking now of our object of analysis, the Cerco neighborhood in the city of Porto has a strong symbology. It is a fragmented and fragmentary territory, in which the logic of advanced marginality (Wacquant, 2007) applies perfectly. It is in the municipality of Campanhã; a territory marked by social and housing problems. The neighborhood is understood as a relational and social space characterized by numerous possibilities of analysis, besides being marked by the presence of several social problems challenging for a sociological investigation (Sousa, 2018).

The OUPA initiative, which emerged under the 'Culture in Expansion' programme, a social intervention project through the arts and promoted by the Porto City Council, was born at a time when cultural policies were valued and used by local governments to disseminate local culture and promote social inclusion. The programme was created in 2013 with the scope of placing and promoting cultural offer throughout the city of Porto, namely in the centers and peripheries. Later, in 2015, the OUPA project was included in the programme and aimed to reconcile working with youth populations, that is, with NEET young people and bring them closer to the arts. However, as the president of the group OUPA! Cerco confessed to us in several informal conversations, no one expected that the young people of Cerco were already close to the arts, needing only an opportunity to make them known and expressed, and 'Cultura em Expansão' and OUPA were that opportunity and hip-hop was the fuse. The program lasted a year in the Cerco neighborhood and included the participation of several national artists.

In the initiative participated Joca, DrunkNigga, Ruubi, Kest, RauneFenix, *Ricardinho*, Lendária Treze and Black Mama^4^. These young people ended up producing two albums, one in 2016, entitled “OUPA!” and the second in 2018, called 'Liquid City', they performed at the Rivoli Theater, and even represented Portugal at a youth intervention festival for the arts in Slovakia. In addition, they welcomed the President of the Republic with a performance in the neighborhood in 2016, something that had never happened before. Due to the success of the programme in the Cerco Neighborhood, the Porto City Council decided to extend it to other social neighborhoods in the city, such as Ramalde and Lordelo do Ouro, but with the end of the artistic residencies OUPA! Ramalde and OUPA! Lordelo ended up extinct, being OUPA! Cerco was the only one that remained active, under the form of an Association, i.e., the E.C.O Cerco Estúdio Comunitário Association.

Initially, our intention was to work only with NEET young people from Porto's Cerco neighborhood; however, as we were planning the activities (graffiti, cypher's, photography, showcase, discussion sessions etc.) we came across a question: if our aim is to promote initiatives aimed at reducing feelings of exclusion, as well as to counter the stereotypes created about the Cerco Neighborhood as an unsafe place, something we have been doing since the beginning of the 20th century^5^ (Guerra, 2002, 2003), why not open up the initiatives to other young people who do not live in the neighborhood? That is what we did. Focusing on the three main activities, namely cipher, graffiti and photography, we started to widen our dissemination process, in the sense that we tried to establish partnerships with several institutions that work with young people in Porto, such as the Campanhã Youth Center. Besides that, we also made an investment to attract local artists as mentors, but from various geographical areas, such as the Cerco district, Vila Nova de Gaia, Rio Tinto, Espinho, among others. Besides that, the process of editing and filming the short film here under analysis, was done in partnership with a student from the Faculty of Fine Arts of the University of Porto, and this project was her first contact with the neighborhood. So, we can refer that we sought to dilute the boundaries between the neighborhood and the rest of the city, making it an open space susceptible to other institutional and non-institutional visions.

Thus, this article is structured in the following way: first we will analyse the methodologies that guided the project 'The Neighborhood is Ours II', based on arts-based research, like cine-worlding or Participatory Action Research; then we will analyse the documentary 'Não vivemos só de sonhos' (Dreaming is not enough)^6^, in which we follow the trajectory of *Ricardinho*, one of the main actors of the mentioned project. In a first part we will analyse how we can establish sociological parallels with the personal approach of *Ricardinho*, one of the main participants in the project, which goes from areas as distinct as urban sociology, with concepts such as advanced marginality, to the sociology of music, with the identity importance of music and, more specifically, how hip-hop is today the soundtrack of many neighborhoods and cities; in a second part we will analyse the impacts of the project, whether at the level of the participants or in the neighborhood.

## From little things big things grow: Art-based research in Cerco's neighborhood

Social inclusion has been one of the most worked topics in sociological terms, however, none of these debates or studies have managed to provide an effective answer regarding its implementation and practice (Felder, 2018), perhaps because the cycles of social exclusion are in constant (re)production. For the preparation of this text, we considered that inclusion refers to the connection of an individual with a certain physical space, but also with a set of interpersonal relationships that take place in that same space (Norwich, 2008). Focusing on arts-based research, we consider that its main advantage is that it promotes the intersection between interpersonal and social levels of inclusion. Let us take as an example the project we propose to analyse here: the arts-based research and intervention fostered the integration of young people and artistic mentors in interpersonal terms (creation of friendships, networking, sharing of tastes and attitudes), as well as enabling the crossing with others distant from those interpersonalities, such as researchers or the university.

Aligned with a cultural turn, we can also speak of a participatory turn in the 1990s: a growing concern, in the art world, with collaborative and communitarian processes, with very clear ideological objects of changing the social situation, whether of a disadvantaged community, a social group, etc. (Bishop, 2006). Artists, especially those with greater ideological concerns, have always sought to warn against social situations that they considered unacceptable. Take the case of the Portuguese neo-realist artists, who depicted the economic and social difficulties of Portuguese workers or farmers during the Estado Novo dictatorship. Nowadays, the big difference is the participatory scope. The intention is still to improve the living conditions of a group or of a specific community, but this time there is a concern with a participatory and non-hierarchical dynamic, in which participation is no longer passive and participants have a shared responsibility at the various levels of the projects. Participants become collaborators and there is a recognition that collective action and social change can only be achieved collectively.

One of the methodologies used is Participatory Action Researchers (PAR). Participatory in nature, it seeks to promote social change through collective participation, where participants are seen as co-researchers with the purpose of promoting personal, organizational and structural change (Brydon-Miller and McGuire, 2009). There are several methods that can be used here, such as poetry, photography or music, in order to help participants “identify, represent, and enhance their community” (Wang and Burris, 1997, p. 389; Brydon-Miller and McGuire, 2009).

Through music, for example, participants can critically address and reflect on the qualities of their community, fostering a critical dialogue among themselves and between themselves and researchers, as well as developing a greater capacity and expertise to communicate these issues with policy makers. Unlike methods such as photography, which ultimately may not involve collaborative work between participants, each collecting and interpreting photographs based on their personal interests, music is a collective social practice that provides people with a sense of belonging, where their aspirations are no longer just individual, but part of aspirations shared by a large number of people (DeNora, 2000). Especially if we refer to popular music, it allows a counter-discourse to be established as an instrument of refutation of dominant ideologies and articulation of new alternatives, allowing participants, as political actors, to leave the periphery of political discourse (Valassopoulos and Mostafa, 2014).

Music allows one to express frustration and articulate it in an instrument that can reach countless people (Côté, 2011). Thus, music creates spaces for discussion, capable of articulating fragmentary ideas and forming a common culture. Drawing on Williams (1989), we can say that music shapes and transforms identities and can be an arena of resistance capable of interacting with the sociopolitical problems that affect communities.

However, Sanders-Bustle (2020) is right that it is important to be careful when introducing methods into PAR. Whether it is music or poetry, for example, they all refer to conventional processes based on institutional structures, histories and systems that can stifle the emergence of new processes and critical visions that aid social change. To avoid this reality, it is crucial that researchers consider the importance of dialogue (Kester, 2011). Dialogue should be understood as a means of challenging the status quo and an enabler for new forms of critical thinking to emerge. Kester posits two key issues: first, dialogue as a detailed understanding of the social context in which participants speak and act; second, an interaction grounded in empathy. In PAR, in this way, dialogue is a means for social change, a mutual negotiation over meaning (Lather, 1986) which leads to reciprocity.

Dialogue and community participation are based on the concept of progressive education postulated by Dewey (1934) at the end of the 19th century. Education, for this pedagogue, is not only the accumulation of knowledge, but a form of civic responsibility and a means to achieve (and maintain) democratic values. Dewey's modernity does not stop there: this author also defended the importance of art in the public sphere. Art, for him, would be a way to communicate across ethnic, classist, nationalist lines, etc., since through art people get to know each other's differences and similarities, breaking down barriers and creating a sense of community. This allows us to address another concept, that of art and community participation. This is a form of collective participation in which researchers and/or artists work directly with local participants to create art that authentically expresses the problems of these communities (Krensky and Steffen, 2009). Forrest-Bank et al. (2016) associates this concept with that of artivism, as artivists unmask the state of democracy, or in other words, exhibit those forgotten by democracy and the state. It is a form of protest and resistance, but also a celebration and the exploitation of what is invisible, be it a group dynamic, a social situation, etc., resulting in an art based on first-hand experience (Bishop, 2012).

As we talked about before dialogue, social interaction and cooperation are at the basis of this process: it is necessary first to develop activities that serve as a catalyst to create feelings of connection and trust between the different individuals who belong to the community, with different social backgrounds and interests. Cineworlding (Macdonald, 2021) is one of the strategies we have developed that is important to address. It is a strategy that has the potential to contribute to strengthening the participatory and collaborative approaches we talked about earlier. The use of filmmaking methods, due to their cost and lack of institutional support, has been low. However, with the ecosystem of digital cinema, which emerged in the first decade of the 21st century, already perfectly established, with cheap digital cameras, smartphones and open-source film editing software, the reason for the timidity in the use of cinematographic methods has ended. Cineworlding recognizes that to make art is to do research and that each film, as a consequence of the need to create a logic at the level of film planning, editing and dissemination, is a machine of collective and communal expression, which goes beyond passive participation or participant observation (Marciniak and Bennett, 2016). Making a documentary implies the development of knowledge, of a know-how, which broadens the pedagogical spaces themselves (Harbert, 2018). Hence the happy conjugation between the application of music and film in these participatory and community research processes. More than that, this approach has the particularity of opening endless future paths. As Macdonald (2021) tells us, each film is a rhizome with various connections that can be continued by others. In this way, each collective film is never a finished work, each film creates the basis for another film and is something that continues to influence and establish connections^7^.

Finally, all these methods raise ethical issues and decisions that are not always easy to make. Sanders-Bustle (2020) speaks of the need for researchers to de-skill, that is, to move away from standard research processes and logic. This is an essential step toward achieving a participatory and collaborative logic. If the researcher puts his/her needs and skills above those of the participants, if the researcher does not put aside his/her concern for authorship, all this will imply a perpetuation of institutionalized structures and put the whole process at risk. Equally, the researcher must recognize the multiplicity of voices and ensure that those voices are heard and included and, furthermore, recognize their position and the limitations that this position implies at the time of research (Staikidis, 2014).

## We shall overcome, or the periphery must conquer the center

From the periphery to Cerco is our tag

Big hug for those who do their own thing

No cliché, no doing what you see on the net

This rap is ours

We for you

That means, the sound is yours

This beat is ours, this rap is ours

We for you

Meaning the sound is yours

This is the view from the Cerco where I am now

Where I try to report to the outsiders

It's not just a neighborhood, it's respect above all

I accept who comes to know this world

Sometimes not everything is what it seems

And reports aren't always real about what happens

But I don't forget reality and we'll take the siege to the center of this city

In my neighborhood or yours

On my street or yours

We'll take what's ours in this raw reality

From the center to Cerco, from Cerco to the center

Cerco marking the movement

(OUPA Cerco, “Do Cerco ao Centro,” 2016, our translation).

In the lyrics and in the documentary, the words “bairro” [neighborhood], “bairrismo” [neighborhoodism] and 'respect' often appear together. This refers to what we talked about at the beginning, the processes of advanced marginality. If we rely on a Simmelian perspective, space has no sociological meaning; it only becomes relevant when we consider the social relations that are developed there, the representations of space that are constructed by the actors. It is necessary to note that the social space conditions enable social interactions and, therefore, it can be considered a space of possibilities. Wacquant (2007) considers that since the 1970s there has been the rise of an advanced marginality, a consequence of factors such as the retreat of the Welfare State. If in the post-war decades marginality was in the neighborhoods of working-class communities, now, and increasingly, this new marginality is concentrated in specific territories isolated from the rest of the city, seen from the outside as places to avoid, where violence, poverty, and crime reign, and where those excluded from society live. This leads to the fact that the inhabitants of these kinds of neighborhoods end up recognizing that living in the neighborhood is a synonym for having a devalued social image, a stigma associated (Guerra, 2003).

This leads us to the “bairrismo” [neighbourhoodism] that *Ricardinho* tells us about. This is an example of the Weberian concept of social closure. The ability of a particular social group to create a monopoly, in this case in urban space, through restricted access, which enables it to access certain rewards, whether monetary or symbolic (Savage and Warde, 2002). This can be turned around in a reality such as a social neighborhood, where a sense of pride in the difficulties is created and an us/them feeling is created, with a population with strong solidarity and inter-knowledge networks (Guerra, 2002). This is fundamental in face of the existing distance from the rest of society.

*Ricardinho* doesn't stop there. As if endowed with a sociological state of the art on urban inequalities, he talks about the difficulties of moving around when living in a social neighborhood. He talks specifically about the feeling of living in a 'gap', of living in a forgotten neighborhood of a forgotten area. This “gap” is 2 fold: before the city center, very distant and almost inaccessible, but even a gap before the nearest neighborhoods since the neighbourhood's connections to the geographically closer areas were few. As *Ricardinho* tells us, “It felt like we were stuck here.”

This reminds us of the classic work of Rémy and Voyè (1992), in which it is discussed that certain social neighborhoods are mono-functional, which causes feelings of social exclusion, due to the importance of mobility in modern societies. During the process of urbanization, cities developed a set of facilities, from schools to hospitals, which led to two phenomena: certain neighborhoods were reduced to monofunctionalism and, on the other hand, urban spaces that concentrated most of the essential facilities emerged. It is no longer possible to ensure all the needs in a neighborhood, which accentuates the predominance of the mobility phenomenon. As mobility depends on capital that the inhabitants of these neighborhoods tend not to have, as well as the absence of a public interest in resolving this situation, this accentuates inequalities and leads to that feeling so well-defined by *Ricardinho* of being in a “gap.” It is also not by chance that the documentary produced during the OUPA project, directed by André Tentugal^8^ and Vasco Mendes^9^, has been called “Cercados” [Surrounded]. In fact, thinking of the specific case of the documentary 'Cercados' [Surrounded] and of our work under analysis here, we can, to a certain extent, gauge that art—especially audiovisual art—is assumed as a mechanism of rupture with this social closure and, even more, it is important to highlight that during the co-collaborative process of building the script of the documentary, this was a topic and an idea defended by *Ricardinho*: art allows us to transport ourselves beyond physical, geographical and social barriers.

The neighborhood then ends up being a place visibly distant from the center, physically and symbolically. However, in *Ricardinho*'s view, through the formal and informal action initiatives of young people and through the arts, it is possible to reconcile the neighborhood with the city center. On the other hand, the center is increasingly seen as a space of power and decision-making, which means that the work—artistic or otherwise—developed by young people like *Ricardinho* is often dismissed or camouflaged. So, in this sense, the realization of participatory and collaborative artistic projects finds its genesis here: the search for a break with such assertions. The city is thus marked by unequal accesses that lead to unequal destinations and that, in turn, define unequal centralities (Guerra, 2002; Hobson, 2019). In the case of young people like *Ricardinho*, art has always been an escape and a weapon of combat. As the song says,

But I don't forget reality and we'll take Cerco to the center of this city/In my neighborhood or yours/In my street or yours/Accept what is ours in this raw reality/From the center to Cerco, from Cerco to the center/Cerco marking the movement (OUPA Cerco, “Do Cerco ao Centro,” 2016, our translation).

The unequal urban reality is, we can say, inscribed in the flesh of these young people and what better way to manifest against it than through hip hop, which is the visible face of the current urban protest (Guerra, 2020). This is the objective embodied in the lyrics, to take Cerco to the city center, to truly describe the reality of the neighborhood, beyond news in tabloids or social networks, but, and this is important, but always with an active role, always as the actors of the situation. If it weren't for “Cerco marking the movement.” Here we have the second objective inherent to the making of the documentary: to show that art, in the hands and voice of young people like *Ricardinho* is, effectively, a weapon. At the same time, the idea that this documentary is based on the development of knowledge is also expressed here, and that it assumes itself as a pedagogical space (Harbert, 2018) because the young people who watch it, in the future, whether they are from the Cerco or not, will be able to perceive that there are alternatives and distinct modes of action.

This also serves to introduce the role of the arts, more specifically community art, in enhancing the social integration of these young people. Again, we would have a lot to gain if we listened more attentively to these actors, especially at the time of building intervention projects. *Ricardinho*, who was 30 years old at the time of the documentary, already has a lot of experience with these yo-yo projects, so to speak. He defends the need for an intervention by the arts, coming very close in his description to what Howard Becker defends, that is, that art is always collective, since we need someone to do something, or, even when we do something alone, if that is possible, we are always inspired by someone. In the end, art doesn't exist in a vacuum.

Returning to *Ricardinho*'s knowledge about the reality of the intervention projects, he does not shy away from launching his criticisms. In effect, if we previously mentioned that one of the aims of documentaries as a research technique was to demonstrate the existence of other realities through sound and image, it seemed to us of utmost relevance to introduce into the documentary all the difficulties and negative consequences of research projects. Acknowledging that something failed and knowing why it failed is also pedagogical, not only for the populations but also for the researchers and, in this sense, it reinforces the importance of adopting participative and collaborative methods in these contexts, in order to ensure that future projects are made with the needs of the populations in mind, in this case the young people. Art has a role as an alternative to practices such as drug trafficking and criminality. But, more importantly, art in the sense of resistance and, as he says, “the capacity to manifest and express, what you can or cannot change in the neighborhood and in a person.” And to be an example for the younger ones, in the sense that you can show them that it is possible to make a life of the arts, even if it is difficult.

However, for this to happen, the research needs to be well set up, as is the need for social projects to choose musical genres that appeal to the tastes of the younger generation, such as hip hop, which is the soundtrack of many cities. In essence, a dissociation between what researchers think young people in the neighborhood like and what young people really want. The problem of top-bottom policies and an absence on the ground and the inability of researchers to assess what is really needed. What makes young people lose motivation. *Ricardinho* talks about the fatigue that social projects provoke. He says that at the age of 30 he has seen countless projects that came to nothing. Hence, he says that although many of the researchers arrive with good intentions, there is already a negative reaction from people.

Moreover, in many cases they are surprised by the acceptance of art by the young, forgetting, or unaware, as *Ricardinho* says, that the young people of the neighborhood were already dedicated to the arts, in the streets of the neighborhood, singing and dancing hip hop.

The urban space is a musical territory, with contradictory discourses and target of struggles, each place, like the Cerco neighborhood, can create narratives resulting from life experiences in that neighborhood (García and Feixa, 2020). If we situate ourselves in the analysis of the lyrics of the songs or the cipher, we find several references to space, namely the neighborhood, the street, and the city. Here, the neighborhood has a double symbology. Firstly, it is the symbol of their exclusion and their living conditions: it is the standard of living that they throw in the face of the society that discriminates against them. Secondly, and paradoxically, it is also reconverted as a space that aggregates their identities and an ontological sense of belonging.

First black of Cerco, king without fear/(…)/Friend of my friend, call me true/(…)/I've stopped in many places, but I don't forget where I come from (OUPA Cerco, “Eu Sou,” 2016, our translation).

The neighborhood is ours/ And so is the street/ Respect is important/ And we have unity^10^

There is, in essence, a reconversion of a non-place into a place of identity through songs and cipher (Fradique, 2003). It is possible to see in these lyrics the search to establish positive collective identities, of a clear link with pride of place. In one of them, particularly interesting, there is the appropriation of all stigmatizing discourses and a reworking, in which the “traffic” (of drugs) is of words and the “bullet” (criminality) is the knowledge.

They're coughing/This is our voice/Today the neighborhood is ours/That's my line please don't cross/Today I'm alert and focused on the movement/My traffic is words/My bullet is knowledge^11^.

We have made who we are/Show where we come from/We know where we are going/United (OUPA Cerco, “Eu Sou,” 2016, our translation).

Above all, we have a culturalization of the political. Young people use hip-hop to establish their (politicized) discourses on reality. Hip-hop serves for young people to position themselves politically, and to politicize is to make oneself audible, to direct oneself toward the center. Willis (1990) notes that youth cultural productions, such as music, serve to have their actions recognized as significant and meaningful material and symbolic practices. For García and Feixa (2020), these are important strategies of de-marginalization. In short, these are strategies of politicization with local meanings. Thus, a genre such as hip-hop is used to demonstrate the stigmatizing situation in which they live. Here hip-hop implies a structuring and structuring axis of the collective identities of these young people.

And this can only be apprehended by applying prevention in action methodologies and arts-based research, since it was the way that allowed us to gather information about the experiences of these young people which, by applying standard methodologies, such as surveys or interviews, would not be obtained. These are crucial perspectives for understanding the condition of NEET and their subordinate position as residents of the Cerco neighborhood. The use of creative methodologies enabled the young people to communicate their messages without fear. The writing of these letters and cipher helped the participants to express what was important to them at challenging moments in their lives.

## A change is gonna come

When we spoke to *Ricardinho* it is possible to understand the choice of the documentary's title: 'We don't live by dreams alone'. It is a continuation of *Ricardinho*'s criticism of many of the social support projects he has seen. What he wants are projects that create skills and trajectories of labor insertion through the arts (Howard, 2022), and then to use these examples as proof that it is possible to live from and through art in a neighborhood like Cerco. But not only that: give people the idea that they can be the actors of individual and collective change.

This is where the OUPA process came in, in 2015, which aimed to reconcile working with young people, bringing them closer to the arts and culture. Basically, it was the reconciliation of an artistic residence with the people who were already making music in the neighborhood. The project, in *Ricardinho*'s words, had the effect of “bringing the pieces together, of making people believe again, of talking about your area, of putting your area on the map. Talking about how we are the same as the rest of the city (…) We managed to prove that when conditions are right, things happen.” The big test, however, is whether the results persist or, on the contrary, if the project is closed, everything goes back to square one?

According to *Ricardinho*'s words, as well as his trajectory, it is possible to gauge that the project created capitals that were appropriated by the actors. First, the project resulted in something that will stay for life, as it implied something physical, with two albums, one in 2016 and another in 2018. These Are associated with a materiality that refers to a memory, of a nostalgia for the future, for a positive moment, a collective moment in which there was an overcoming (Boym, 2002). But we cannot only look at it as a 'dead' object. That is, the project had ramifications. After the performance at Rivoli, in 2015, they were invited to two major Portuguese festivals, Marés Vivas and Sudoeste; they then performed at Maus Hábitos and were chosen as the Portuguese representatives in a festival in Slovakia.

It was one of the 'seeds' that Ricardinho tells us about. They began to be present in the media and, consequently, to receive invitations to perform. Thus, the performance was the end of the artistic residency, but the effects continued and they became the center with politicized speeches about the reality of social neighborhoods.

The albums, for example, represented an effort of professionalization and autonomy of the young people and they were responsible for all the creative decisions. It is curious the choice of the name of the second album, “Liquid City,” a clear homage to Paulo Cunha e Silva, councilor for Culture of the Porto City Council who died in November 2015 and responsible for the programme Cultura em Expansão, within which OUPA was created. This is not the only tribute: the walls of the association's headquarters are another tribute to Paulo Cunha e Silva: on one side, the quote “The future is today” and, on the other, “Making the city of Porto a liquid city.” And here is where OUPA's idea is encapsulated: that everything is part of the city, neighborhoods, streets, islands, deprived areas.

Associativism was another reality frequently mentioned by *Ricardinho*. That is, the absence of this in the Cerco neighborhood and here we can see another influence of the project. After finishing the OUPA project, several of the young people decided to open an association in the neighborhood, E. C. O. Cerco—Estudo Comunitário Oupa! Cerco, which aims to contribute to a new dynamic in the neighborhood and the area. To do this, they set about building a community studio, firstly, to record their music, and secondly, to work on the social side through the arts. Basically, to perpetuate the legacy of the project, to use themselves as examples, or, as *Ricardinho* told us: “it's not about being on stage singing for a thousand people, but the day that you're giving to children and young people. And knowing that we have many children and young people to work with, not only from the Cerco neighborhood.”

And so far the association has shown vitality: they have held a festival, the OUPA Acampanh'Arte Festival, using DIY strategies (Bennett and Guerra, 2019; Guerra, 2020) that they developed during the project, and have thus created a place to expose the music that is being made in Campanhã, so that the young people they work with have a place where they can perform and expose their music and to “feel the stage,” as *Ricardinho* tells us. A particularity of this festival, as its name indicates, is that it extends to the whole area of Campanhã: it is, therefore, a step toward leaving that “gap” that *Ricardinho* tells us about. After the end of the project, after all the attention that came from the first album recorded, it is important to keep up the fight to create links between the neighborhood and the city center.

All of this, albums, creation of festivals, performances, the association, etc., is the result of the development of know-how and skills that the project implied. Basically, to give a more professional face to what they were doing before the project, which was to rhyme through the streets of the neighborhood (Guerra, 2022a). It was a leverage, it was getting to know some of the ins and outs of the artistic world, because, as Joca, another of the project's participants, admits: “We were completely apart of this world” (in Gerador, 2021).

On the other hand, given the positive impact of the project, the association is regularly invited to give conferences in schools and colleges and they are several times mentioned in the media as a good example of social support measures that have worked (Gerador, 2021). Similarly, one of the proofs of legitimization was the choice of these young people to perform during the visit of the President of the Portuguese Republic, Marcelo Rebelo de Sousa, to the Cero neighborhood. It was *Ricardinho* himself who, after the performance, spoke to the president and did not fail to highlight his great concern center/periphery and began his speech saying: “From the periphery to Belém.”

Despite all the positive points, there are still bumps in the road. In addition to what we mentioned before, the association continues to collaborate with other social projects, such as the CANVAS project and URBINAT^12^. The CANVAS project is based on a logic of prevention-in-action and aims to reduce stigma and feelings of insecurity in socially disadvantaged contexts, among NEET young people, using the transformative potential of the arts. URBINAT, on the other hand, focuses on the link between urban regeneration and social integration. The interventions of this project focus on public spaces and co-creation, with the aim of fostering new social relationships and social change. In other words, the association is starting to be seen as a key and experienced intermediary in this type of arts-based interventions and a gateway to gaining the trust of young people in the neighborhood.

However, there are still problems, as *Ricardinho* tells us, despite framing them in a logic of 'resilience' (McRobbie, 2018). In addition to the novelty that was opening and working in an association, everything that involves at management level, the association stopped receiving financial support and the space that they gave to the association had been closed for many years and needed a remodeling, and as they wanted a quality space to receive the youngsters, this implied personal financial costs and the use of DIY strategies: they were the ones who made the tables and chairs, who padded the sofa, etc. It's the “setbacks that make you reinvent.” Then, the lack of support does not allow them to dedicate themselves full-time to the association, having to combine it with other work, a reality very characteristic of the Portuguese creative industries context (Guerra, 2017, 2018). One of the criticisms is that there is not always a dialogue between the new social projects and this association. Basically, the old problem of projects planned from the outside and without communicating with the actors in the neighborhood, even with a proven association. Basically, the criticisms that we mentioned earlier, that social projects are not always designed to leave 'seeds'.

## Fight for the power

I am different, just like you/I am also people, also from the neighborhood (OUPA Cerco, “Eu Sou,” 2016, our translation).

Having said this, and taking into account *Ricardinho*'s words in the documentary, as well as the lyrics and cipher produced during the project, we have proof of the cultural importance of popular music, in this case hip hop, for the youngest (Bennett, 2000). An issue, as we have seen, that *Ricardinho* and many others had been preaching in the desert upon the arrival of yet another social project imposed from outside. If we look at some of the excerpts that we have been exposing in the article, as well as those that are possible to listen to in the first album, it is possible to understand that it was through these lyrics that the young people exposed their emotions and feelings which, in another context, such as the always asymmetric interview context, could be repressed (Baker and Homan, 2007; Guerra, 2019, 2020). Even more so when we talk about young people coming from the Cerco neighborhood, perhaps the most stigmatized neighborhood in Porto. It is important for researchers to be aware that young people are not alienated, on the contrary, what we found was that these young people are well-aware of the processes of stigmatization they undergo (they can feel it when they apply for jobs or go to a different school), but they are also more than prepared to challenge them and to opt for new forms of citizenship and the conquest of a space within the urban area and in society (Guerra, 2020).

However, it is important to mention the difficulties that were experienced throughout the project. As we discussed in the second section, it is important to have a deskill on the part of the researchers. It is not always easy to do so, however. The main need for a deskill relates to the language itself. There is a gap between the dialogue of young people and that of researchers. The language is the same, but there were times when it seemed that the sides did not understand each other, and it took continuous adaptation by the researchers to break down this boundary that prevented a fluid dialogue without hierarchies (Sanders-Bustle, 2020). Another difficulty was at the very beginning, upon arrival in the field, when we found it difficult to attract young people to this project. As *Ricardinho* explains in the documentary, it is the problem arising from the tiredness of countless social projects that arrive, create illusions, end and leave everything the same. A tiredness that hides some humiliation on the part of the young people, who feel that they have almost been used and see no improvement.

Hence the importance of the arts in general and popular music in particular, i.e., providing a context for young people to use art to resist and exist. The purpose, therefore, is to create a critical and transformative ethos and praxis.

## Data availability statement

The raw data supporting the conclusions of this article will be made available by the authors, without undue reservation.

## Ethics statement

Written informed consent was obtained from the individual(s), and minor(s)' legal guardian/next of kin, for the publication of any potentially identifiable images or data included in this article.

## Author contributions

Both authors listed have made a substantial, direct, and intellectual contribution to the work and approved it for publication.

## Funding

This article is part of the development of the CANVAS project—Toward Safer and Attractive Cities: Crime and Violence Prevention through Smart Planning and Artistic Resistance. CANVAS was financially supported by the European Regional Development Fund (ERDF) through the Competitiveness and Internationalization Operational Programme COMPETE 2020 and the project funding POCI-01-0145-FEDER-030748. In addition, the publication was supported by FCT—Foundation for Science and Technology, within the scope of UIDB/00727/2020.

## Conflict of interest

The authors declare that the research was conducted in the absence of any commercial or financial relationships that could be construed as a potential conflict of interest.

## Publisher's note

All claims expressed in this article are solely those of the authors and do not necessarily represent those of their affiliated organizations, or those of the publisher, the editors and the reviewers. Any product that may be evaluated in this article, or claim that may be made by its manufacturer, is not guaranteed or endorsed by the publisher.

## Footnotes

^1^The Cerco do Porto Neighborhood was built in the 1960s, in order to meet the housing needs of the population; however, during the 1980s and later in the 1990s, it became an invisible space, which was associated with discourses inherent to stigmatization, criminality, violence and security. It has already been the target of several political interventions, however, these did not have effective long-term effects, making it still seen today as one of the most problematic spaces of the city of Porto.

^2^*Ricardinho* aka Ricardo Lopes has always been interested in artistic practices, from dance to theater and music. He currently works as a promoter of the Bairro, being involved as a partner in several artistic and social intervention projects, thus passing on his testimony about his journey as a NEET youngster. For more information about his journey, please see here: https://www.publico.pt/2021/01/10/sociedade/noticia/ricardinho-jovem-nemnem-viu-hip-hop-alavanca-1945659?fbclid=IwAR1XwztDycRbEalP7H1OqcFBoFJJVhnfxDy6PIbWbyuMVKTA70oCPCDNmts.

^3^OUPA! is an initiative of Municipality of Porto, integrated in the project “Culture in Expansion,” which consists of a four-month artistic residence with young people from the Cerco do Porto Neighborhood and is developed by rapper Capicua, Gisela Borges, André Tentúgal, Vasco Mendes, Diego Sousa, Tiago Espírito Santo, Pedro Cruz and Pedro Nascimento. OUPA! is an empowerment process that aims to stimulate the DIY spirit and strengthen the cultural identity and self-esteem of young people in the Eastern area of Porto through word and music, allowing the dilution of cultural and experiential barriers between the neighborhood and the city, giving visibility to the territory and enhancing the sense of belonging of the participants. More information at: https://www.facebook.com/oupacerco/about/?ref=page_internal.

^4^These are the artistic names of the participants.

^5^This relationship with the neighborhood and its population was established in the 1990s, when one of the authors of this article (Guerra, 2002) carried out her research work leading to her Master's degree. Later, in 2018, the second author of the article (Sousa, 2018), also carried out her master's dissertation in the same geographical context, thus setting the motto for the realization of the workshops “The Neighborhoods is Ours!” (2019–2021).

^6^Available online at: https://www.youtube.com/watch?v=kJzvticxcJY&ab_channel=ProjetoCANVAS.

^7^For the making of the short film we counted on the informed consent of Ricardo Lopes and the other participants according to the normative of the American Sociological Association.

^8^André Tentugal is a Portuguese cinematographer and director.

^9^Graduated in Cinema at the University of Beira Interior, since his first short film his work crosses music, rhythm, architecture and cinema. Besides videoclips for emerging Portuguese bands, he has created documentaries, where the main theme is music, the city and the people, where the main theme is the music, the city and the people. Besides Portugal, he has filmed in different countries such as France, Germany, United States, Poland, China and United Kingdom.

^10^Chorus of the cypher made by young people during a workshop. For more, see Guerra (2022).

^11^Improvisation by an youngster during a project workshop.

^12^URBiNAT focuses on the regeneration and integration of underserved city districts. Project interventions focus on public spaces and the co-creation, with citizens, of new social and nature-based relations within and between different neighborhoods. Using a holistic approach, taking into account the full physical, mental and social well-being of citizens, URBiNAT aims to co-create a Healthy Corridor as an innovative and flexible nature-based solution (NBS), which itself integrates a large number of micro NBS emerging from community-driven design processes.
